# Utilization of insecticide-treated nets by under-five children in Nigeria: Assessing progress towards the Abuja targets

**DOI:** 10.1186/1475-2875-7-145

**Published:** 2008-07-30

**Authors:** Olusola B Oresanya, Moshe Hoshen, Olayemi T Sofola

**Affiliations:** 1 National Malaria Control Programme, Federal Ministry of Health, 2nd Floor, Yobe House, First Avenue, Off Shehu Shagari Way, Maitama, Abuja, Nigeria; 2 Hebrew University – Braun School of Public Health and Community Medicine, Jerusalem, Israel; 3 National Malaria Control Programme, Federal Ministry of Health, 2nd Floor, Yobe House, First Avenue, Off Shehu Shagari Way, Maitama, Abuja, Nigeria

## Abstract

**Background:**

The Abuja target of increasing the proportion of people sleeping under insecticide-treated nets (ITNs) to 60% by the year 2005, as one of the measures for malaria control in Africa, has generated an influx of resources for malaria control in several countries in the region. A national household survey conducted in 2005 by the Malaria Control Programme in Nigeria assessed the progress made with respect to ITN ownership and use among pregnant women and children under five years of age since 2000. The survey was the first nationally representative study of ITN use assessing progress towards the Abuja target amongst vulnerable groups.

**Population and Method:**

A cross-sectional survey of a sample of 7,200 households, selected by a multistage stratified sampling technique from 12 randomly selected states from the six geopolitical zones of the country. Data collection was done during the malarious rainy season (October 2005) using a modified WHO Malaria Indicator Survey structured questionnaire about household ownership and utilization of mosquito nets (treated or untreated) from household heads.

**Results:**

Household ownership of any net was 23.9% (95% CI, 22.8%–25.1%) and 10.1% for ITNs (95% CI, 9.2%–10.9%). Education, wealth index, presence of an under-five child in the household, family size, residence, and region by residence were predictive of ownership of any net. The presence of an under-five child in the household, family size, education, presence of health facility in the community, gender of household head, region by residence and wealth index by education predicted ITN ownership.

Utilization of any net by children under-five was 11.5% (95% CI, 10.4%–12.6%) and 1.7% (95% CI, 1.3%–2.2%) for ITN. Predictors of use of any net among under-five children were fever in the previous two weeks, presence of health facility in the community, caregiver's education, residence, and wealth index by caregiver's education; while religion, presence of health facility and wealth index by caregiver's education predicted the use of ITN among this group.

**Conclusion:**

This study demonstrated that the substantial increase in ITN utilization among children under five years of age in Nigeria is still far from the Abuja targets.

## Background

Malaria represents about 1.4% of the global burden of disease [1] and in Africa, it is the primary cause of disease burden as measured by Disability Adjusted Life Years (DALY) lost of 10.8% [2,3]. The continent bears over 90% of the global burden of about 2.7 million deaths attributable to malaria; and houses over 300 million people who suffer from this disease yearly, the worst hit being young children and pregnant women [2-4].

More than three quarter of global malaria deaths occur in under-five children living in malarious countries in sub-Saharan Africa (SSA) [5], where 25% of all childhood mortality below the age of five (about 800,000 young children [6]) is attributable to malaria [2]. Of those children who survive cerebral malaria, a severe form of the disease, more than 15% suffer neurological deficits [4,7], which include weakness, spasticity, blindness, speech problems and epilepsy. Where such children are poorly managed and do not have access to specialized educational facilities, these deficits may interfere with future learning and development [5].

About 30–40% of all fevers seen in health centres in Africa are due to malaria with huge seasonal variability between rainy and dry seasons. At the end of the dry season, it is less than 10% and more than 80% as the rainy season winds up [8].

In Nigeria, malaria is the leading cause of under-five mortality contributing 33% of all childhood deaths and 25% infant mortality. As a child will typically be sick of malaria between 3–4 times in one year, the disease is a major cause of absenteeism in school-aged children, thus impeding their educational and social development [5] and subsequently robbing the country of its future human resources.

Several global and regional attempts have been made at controlling the disease in the past with little success as a result of ineffective strategies used and insufficient resources. However, the most recent launching of the Roll Back Malaria initiative has generated a lot of resources for the control of the disease with simple and cost-effective interventions, with a special focus on the most at risk. At the historic Malaria Summit hosted by Nigeria in 2000, African Heads of States made a declaration to halve the burden of malaria by the year 2010. One of the targets set for the first five years was to ensure that the vulnerable groups, children under five years of age and pregnant women, have access to and sleep under insecticide-treated nets (ITNs) [9].

Several studies have demonstrated the efficacy of ITNs [10-18]. Between 1986 and 2002, at least 81 trials and over 30 descriptive studies carried out in every type of malaria setting worldwide have documented the positive impact of ITNs on child and adult morbidity and mortality [13]. Most of these studies were summarized by Lengeler in 2004. He found that in five randomized controlled trials (RCTs) an overall reduction in child mortality of 17% could be demonstrated, with six lives saved per year for every 1,000 children protected. The use of ITNs in areas with stable malaria reduced the incidence of uncomplicated episodes by 50% compared to areas where nets were not used, and 39% compared to areas were the nets were untreated. ITNs also impacted on severe malaria, parasite prevalence, high parasitaemia, splenomegaly and improvement in haemoglobin levels of children [18]. A reduction of 27% in child mortality was also demonstrated in an ITN social marketing programme in Tanzania [12,13]. This overwhelming evidence of the efficacy of utilization of ITN was the basis of its adoption as one of the four global Roll Back Malaria (RBM) strategies for malaria control [2].

### Ownership versus utilization of ITN

Two important RBM indicators for monitoring progress towards the set target are the proportion of households which own one or more nets and the proportion of under-five children who sleep under a net [19]. Net ownership is important to assess the effectiveness of the distribution channels of the RBM programme and suggest programme modifications where there are lapses. However, utilization is the crucial indicator that will generate the desired epidemiological impact [20].

Few studies have examined the difference between the two indicators. A meta-analysis of household surveys on net utilization and ownership found a wide gap between net possession and use. ITN ownership was found to be between 0.1% and 28.5%, while use among children less than five years of age ranged between 0% and 16% [21].

Equality is a major issue in ITN ownership. Net ownership has been found to be lowest among the poorest households [22]; thus possibly linking possession to the cost of the net [23]. Authors of a study conducted on the effect of lowering tariffs on nets and netting materials predict that reducing tariffs on insecticides and ITNs from 42% to 0% and the tariff on netting materials from 40% to 5% would increase demand for ITNs by 9–27% [24]. Wiseman *et al *reported a significant association between good access roads to the community and net ownership [25]. Perceived risk of malaria and benefits of the nets by the population also drive demand. Onwujekwe *et al*, in a Nigerian study, found that households with a recent attack of malaria and those with higher willingness to pay were more likely to purchase a net than their counterparts [26]

Utilization has, however, been found to vary with seasons of the year and acceptability of the nets in terms of size, colour and shape. Binka *et al *showed that the time of the year during which the nets are delivered affects use. 99% of the net recipients were found to use the nets during rainy season, while only 20% used it during the dry season [27]. Demographic characteristics like age, education, size of household, and ethnicity also influence use of bed nets. Some studies show that children are less likely to use nets [28,29], particularly in rural areas [22], while others found no significant association between age and net use [30].

Few studies have documented household net coverage and utilization in Nigeria. The 2003 National Demographic Health Survey reported a 12% household ownership of any net and 2% of ITN. Under-five utilization of ITN was 1.2% while 5.9% of them used any net [31]. NetMark, a United States Agency for International Development (USAID)-funded project, conducted a study in 2004 and compared findings with an older one done by the same organization in 2000 in five states in the country. Overall household ownership of any net was found to have more than doubled, from 12% in 2000 to 27% in 2004, while ITN ownership increased from 0 to 9%. The study also documented an increase in utilization of ITN by under-five children compared to previous years to 3.3% [32]. In both studies, rural households were more likely to have a net than urban households.

The President of Nigeria launched an ITN Massive Promotion and Awareness Campaign (IMPAC) and made provision of free ITNs for the vulnerable groups a priority on commencement of implementation of the RBM programme. With support from international donor agencies, ITNs (either re-treatable nets bundled with re-treatment kits or long-lasting insecticidal nets) were procured and distributed.

ITNs were given at immunization posts to children completing their immunization schedule; during stand-alone ITN campaigns in specific rural local government areas; and by means of the Expanded Programme on Immunization, EPI-plus (giving measles vaccine plus ITN) in tandem with supplemental immunization campaigns to saturate the population with the nets in line with WHO's recommendation [33].

The huge cost implication of the Abuja Declaration and the limitedness of available resources [34-36] coupled with the enormous pressure to meet set targets within limited-time spans puts a prerogative on sound monitoring and periodic evaluation. This is crucial for identifying gaps in programme implementation or areas where modifications in specific technical strategies may be needed, where resources should be focused, assessing progress or otherwise and providing feedback to inform future planning [37].

In addition, the United Nations Millennium Development Goals (MDGs) 4, 5 and 6 are directly linked to malaria control, while 1 and 2 are indirectly related. The achievement of the MDGs depends, therefore, on the success of the RBM initiative. An assessment of the country's performance over the first five years of implementation would also give an indication of where the country is with regard to achieving the MDGs [38].

Since the declaration was made, studies have been done to evaluate coverage, acceptability, willingness to pay for and effectiveness of all the interventions in Nigeria [31,32,39-41]. However, only one of those which studied utilization of ITN analysed nationally representative data [31]. The sampling methodology used for the selection of the states included in the NetMark study was purposive, selective and not random; the sample was drawn only from areas where the agency was active with intensive distribution of nets and provision of targeted subsidies for the vulnerable groups and only households with under-five aged children were included in the survey. This selection bias affects the external validity of the study and makes it difficult to generalize the results to the whole country as positive results are likely to be overestimated.

Although the NDHS survey was a larger study, it was carried out two years before the due time for the mid-term evaluation of the Abuja Targets. The results are, therefore, not likely to reflect the current coverage of ITN among children under-five.

To inform policy and re-engineering of programme delivery to meet the set targets, evaluation should not just be limited to percentage coverage of ownership and use. It is important to investigate the predictors of utilization of ITN in a national survey. The two studies described above also failed to examine these associations being limited by the study design, which was descriptive rather than analytical.

This study aims at bridging the current information gap on the status of implementation of the Abuja targets in Nigeria as regards the ownership and utilization of ITNs among the children under the age of five years and determining the factors that predict utilization. This will help identify gaps in programme implementation and provide a scientific basis for policy decisions on scaling up interventions where necessary.

## Methods

### Study area and population

The study was conducted in Nigeria, where malaria is endemic and transmission is stable all year round, with peaks during the rainy seasons which varies by regions; March-November in the south and July-September in the north. The population is huge (about 140 million) and culturally diverse with about 250 ethnic groups and 521 languages. However, the dominant tribes are Yoruba in the south-west, Ibo in the south-east and Hausa and Fulani in the north. The country is divided into six geopolitical regions namely; south-west zone (SWZ), south-east zone (SEZ), south-south zone (SSZ), north-west zone (NWZ), north-east (NEZ) and north-central zone (NCZ). About 70% of its population live in rural areas, where the predominant occupation is peasant farming and standard of living relatively poor. About 20% of the population are aged 0–5 years.

### Data collection

Primary data collected in October 2005, during a national household survey on mid-term evaluation of the Abuja targets in Nigeria, was utilized for the analysis. A multistage stratified cluster sampling technique was employed at national, zonal, state and Local Government Areas (LGA) levels.

The sampling frame, which consisted of all the 36 States + Federal Capital Territory, was divided into six strata (geopolitical zones) and two states from each of the country's six geo-political zones were randomly selected. In each selected state, three senatorial districts were chosen, from which one LGA each was randomly selected (one urban and two rural areas). At the LGA level, all wards were listed and grouped into communities with and without health facilities, and one community each was chosen at random from the communities with and without health facility. A fixed number of households, 100 per community (total of 7,200), was imposed on the design.

Households were randomly selected by spinning a bottle in front of the market/community leader's house and starting from the direction in which the bottle pointed. Interviews were conducted by trained interviewers in the local languages using questionnaires adapted from the WHO Malaria Indicator Survey (MIS) tools.

The household head (usually male) was interviewed for all information about the household, including net ownership and utilization, while the wife, or any eligible woman (aged 15–49 years) in the household was asked about her reproductive history and fever episodes among her under-five children if any. Where there was more than one eligible woman in the house, the one with under-five children was interviewed.

There was no information on the required sample sizes for the women and children in the sampling methodology available from those who conducted this survey. However, using a design effect of 1.25 [42], requiring a 95% confidence interval (CI) length of 1% and a known ITN coverage of 1.2% for children under five and 1.4% for women aged 15–49 years, WINPEPI [43] gave a required sample size of 2,277 and 2,651 for children under five and women aged 15–49 years respectively. The sample sizes for the two groups-6,390 eligible women and 3,585 eligible children-were, therefore, found to be adequate. To detect an acceptable difference of 0.5% in household ownership of nets, with a known current coverage of 2%, the required household sample size is 3,764; however, 5,588 households participated in the survey.

Eligible children were defined as those who are under five and who stayed in the household the night before the interviewer's visit (*de facto children*), while eligible women were those who were in the specified age group (15–49 years) and who stayed in the household the night before the interviewer's visit (*de facto women*). A mosquito net was defined as an ITN if it was pre-factory-treated or has been dipped in insecticide within the last 6–12 months.

### Analysis

Principal Component Analysis (PCA) [44] was used to develop wealth indices for the households based on ownership of durable assets including radio, television, telephone, refrigerator, bicycle, motorcycle/scooter and car/truck. Ownership was coded as 0 or 1 and missing cases were excluded. The households were then divided into socioeconomic quartiles based on their scores. In order to capture wealth differences between urban and rural residences, PCA scores were generated for the two areas separately (urban and rural household wealth indices) and for the combined data as well. The first dimension of the PCA was taken as the score for the household. Cronbach's alpha was calculated to test consistency-reliability. Infrastructural variables were not used to avoid 'urban bias' that could prevent comparism between rural and urban wealth indices; since urban areas are more likely to have higher quality of building and amenities. Internal coherence was tested comparing asset ownership by socioeconomic groups (quartiles) and this gave a clear trend of increasing asset ownership from lowest to highest quartiles. The distributions of scores for the three groups showed little evidence of 'ceiling' or 'floor' effects as they followed normal curves; suggesting appropriate and sufficient choices of variables.

Univariate and multivariate analyses addressed ownership and utilization of any net and ITN. On the univariate level, frequencies and proportions were calculated for household ownership and under-five utilization of any net and ITN, and cross-tabulated with background characteristics of the households and demographic characteristics of the children. Pearson's Chi squared test was used to determine association with a P-value of < 0.05 accepted as significant. Fisher's exact test was calculated for borderline significance and for cells with counts less than five. Logistic regression models were used to determine the predictors of household ITN and any net ownership separately. A model was developed for the combined data in which variables that were significantly associated with net ownership at the univariate level were used as covariates. Data was split into two regions, north and south, and separate models were developed to investigate the predictors of net ownership in the two regions.

Regression models were also used to determine predictors of utilization. A model was developed for the combined data and then split by residence to generate output for urban and rural areas separately. Interactions between region and residence, wealth index and household heads gender, and religion and region were also explored for net ownership and utilization. Odds ratio and 95% confidence interval, CI, were generated for the final models and p-value of < 0.05 for Wald's statistic accepted as significant. The logarithmic transform of the variable 'family size' was used to normalize the distribution before inclusion in the model and all missing data were excluded from the analysis.

The prevalence of fever episodes in under-five children was analyzed on a univariate level by background characteristics and presence of health facility in the area of residence. Fisher's 95% CI was then calculated for the rate ratios and an overall p-value generated.

## Results

### Background characteristics of study population (Additional File 1)

Of the 5,588 household surveyed, 21% were from the SWZ, 14.4% from SEZ, 13.5% from SSZ, 17% from NWZ, 13.2% from NEZ and 20.9% from NCZ. 36% were urban and 64% rural (ratio 1:2). About two thirds of the households were sampled from communities with health facilities. Overall, distribution of wealth was unequal with slightly higher proportions in the upper two quartiles of the combined data, and the lowest two quartiles of the urban and rural data respectively.

In the combined data, wealth index also varied within and between the zones. In the southern zones (SWZ, SEZ and SSZ), a majority of the population fell within the upper two quartiles (>50%) while the northern zones have more households in the lower two quartiles (54% in NWZ and 76% in NEZ), except NCZ where more than 57% are also in 3^rd ^and highest quartiles, the largest proportion (33.5%) being in the 3^rd ^quartile.

Of the 4,625 households with valid data on household religion, 3,044 (65.8%) were Christian, 1,529 (33.1%) Muslim and 52 (1.1%) practiced other forms of religion. A majority of the households in the southern zones (SWZ, SEZ and SSZ) were Christians; the NEZ was largely Muslim (73%), while the ratio of Muslim to Christian households in NWZ and NCZ was about 1.2:1.

Males headed 93% of the households while females headed 7% of them. Generally, female family heads are commoner in the south (ranging from about 9% to 16%) than the north (1% – 3%). Overall mean family size was 5.4 (SD 3.0) ranging from 4.7 (SD 2.4) in SWZ to 6.5 (SD 3.4) in NWZ (Additional File 1).

15.3% of household members were children under-five and 14.5% were 5–14 years old. Age group 50 and above constituted 5.6%, typical for a developing country.

### Univariate analysis

#### Household net ownership

Overall household ownership of any net was 23.9% (95% CI, 22.8%–25.1%) and 10.1% (95% CI, 9.2%–10.9%) for ITN (Additional file 2). A significantly (p < 0.0001) larger percentage of rural dwellers (22.8%) owned any nets compared to urban dwellers (18.3%), and were more likely (p < 0.0001) to own more than one net (11%) than urban dwellers (0.6%). Net ownership varied very significantly (<0.001) by region. Households in SSZ consistently own the least nets in all categories while NEZ households own more nets than other regions, except for more than one net, which is commoner in the NWZ (35.4%). However, information about net ownership was only available from 58% of the households in the NEZ, which could have resulted in an overestimate if the valid cases were more likely to own nets than the missing cases. Also, the SEZ has the 2^nd ^largest proportion of households with any net and the 3^rd ^largest for more than one net. Since the population in the sample from SEZ was mostly rural (98%, possibly attributable to an error in data collection) and rural households were more likely to own nets than urban households, this could have caused an overestimate of the true proportion for the zone.

When household net ownership by region (dichotomized N/S) was stratified by residence (urban or rural), residence was found to modify the association between region and ownership of any net. Rural households in the north were one-and-a-half times more likely to own nets than urban households in the same region (59.5% vs. 40.5%, RR, 1.47, 95% CI, 1.25–1.74), and in the south, about four times more likely than urban households (79.8% vs. 20.2%, RR, 3.94, 95% CI, 3.17–4.91) and this was statistically significant (p > 0.0001). There was no synergism according to the additive model.

These findings were similar for ITN ownership, with the rural households in the north almost twice as likely to own ITNs as the urban households (62.7% vs. 37.3%, RR, 1.67, 95% CI, 1.29–2.19) while rural households in the south were twice more likely to own ITN than their urban counterparts (67% vs. 33%, RR, 2.01, 95% CI, 1.52–2.73). No evidence for synergism was found on the additive model.

Using the wealth index generated for all households irrespective of their urban or rural status (combined), households that fell in the highest quartile were hardly more likely to own any net (26.3%) compared to the lowest quartile (24.1%) with rate ratio, RR of 0.91 (CI, 0.79–1.07); while the poorest households were more likely to have more than one net (13.3%) compared to the richest (10.0%) with a significant RR of 1.3. There was no significant difference in ownership of at least one ITN between the rich and the poor (p = 0.05) and though poorest households were more likely to own more than one ITN than those in the other quartiles, this was significant only for the 3^rd ^quartile (RR 1.5, CI, 1.03–2.21).

Using wealth index generated for urban and rural areas separately, the scenario was similar for urban households and that of the combined data. More households in the lowest group owned any compared to the 2^nd ^and 3^rd^, but those in the highest owned more nets than any other group (p = 0.003). When urban wealth index was dichotomised, the rich households (upper two quartiles) owned significantly (p = 0.045) more nets (22%) than the poorer households (lower two quartiles; 18%).

By rural wealth index, the 2^nd ^quartile households had the largest proportion of nets in all categories. In the category for any net, this was followed by the highest quartile and the lowest had the least proportion of households with nets (<0.0001). When dichotomized, the richer households (upper two quartiles) were more likely to have nets (28.2%) than the poorer (lower two quartiles) households (23.2%).

### Multivariable analysis

#### Ownership of any net

Variables that were significantly associated with net ownership at the univariate level; region, residence, and household wealth index; and other covariates; presence of at least one under-five child in household, religion, gender and age of household head, family size and presence of health facility in the community; were entered in an unconditional logistic regression model (combined Model A) to determine predictors of household net ownership using the stepwise forward likelihood ratio.

In the initial model, combined wealth index, residence, region, religion, household head's gender, presence of at least one under-five child in the household were significantly associated with net ownership; however, when 'presence of an educated eligible woman in the household' as a proxy for household education status, and interaction terms for region by residence, religion by region, and combined wealth index by household head's gender, were entered into the model, the final model did not include religion, however, education and family size became significant.

The odds of a household owning a net if there was an educated eligible woman in the household were 30% higher than a household without an educated eligible woman (p = 0.005). A unit increase in family size increased odds of ownership of any net more than twice (p < 0.0001) while controlling for all other variables; and if a household had at least one under-five child the odds of owning any net was about 60% higher than households with no under-five child (adjusted OR, 1.60, CI, 1.40–1.90). Living in a rural area raised ownership odds by 26% compared to urban residence (p = 0.021), and there was interaction between residence and region with a more than 50% increase in the odds of owning a net if the household was in the north and rural compared to urban households in the south (p = 0.024). Every unit rise in wealth index was found to increase ownership odds 1.24 times (CI, 1.15–1.34).

Data was split by residence and separate models were developed for urban and rural areas (Models B and C respectively) and outputs were generated for the north and south regions separately to identify predictors for the regions. Additional File 3 shows the adjusted odds ratios for the final variables in the different models.

In the final model for the urban region (Model B), the presence of an educated woman in the household raised the odds of owning a net by 42% in the north compared to those without (p < 0.0001), while this was not predictive in the south controlling for other variables in the model. The odds of owning a net were almost three times higher for households with a health facility in their community in the south than those without (OR, 2.88, CI, 1.54–5.37); while they were more than one-and-a-half times lower in the north (OR, 0.59, CI, 0.41–0.81), controlling for other variables in the model. Also, in the north, a unit increase in urban wealth index independently increased the odds of net ownership by 28% (OR, 1.28, CI, 1.09–1.50), a difference not found in the South. An under-five child in the household was very significantly predictive of net ownership in the south with an OR of 3.24 (CI, 1.99–5.27) but not in the north.

For rural areas (Model C), presence of under-five child in the household and health in the community, family size, presence of health facility in the community, wealth index, education and religion were predictors of ownership of any net in the south while only family size and religion were important for net ownership in the north.

Households with at least one under-five child was 1.7 times (p < 0.0001) as likely to own any net as those without, and those with a health facility in their community were 1.6 times (p = 0.001) more likely to have nets than those without health facilities, controlling for other variables in the model. The odds of net ownership increased by more than three-and-a-half in the south, and more than two-and-a-half in the north, for every additional family member (p > 0.0001 and 0.002 respectively).

Christians were more than twice more likely to own nets in the south (OR, 2.35, CI, 1.32–4.20) and twice less likely to in the north than Muslims (OR, 0.23, CI, 0.03–0.91). A unit rise in wealth index and having an educated woman in the house independently raised the odds of net ownership in the south by 33% and 55% respectively, with no effects in the north.

#### Ownership of ITN

Similar logistic regression models were developed in which the dependent variable was ownership of at least one ITN by the household. Variables analysed on the univariate level were adjusted for potential confounders in a combined model (Model I) and the data was then split by residence (urban, Model II/rural, Model III) and outputs generated for the north and the south (Additional File 4).

In the combined model, although region and combined wealth index were significant on the univariate level, when entered into the model with other covariates, including presence of an under-five child in the house, presence of an educated eligible respondent in the household (as a proxy for education status of the household), health facility status, gender of household head, residence and interaction terms for region by residence, education by combined wealth index and combined wealth index by household head's gender; these two variables were not significantly associated with household ITN ownership. The single variables in the final model were under-five child in the household; health facility status, household head's gender, education status and family size and these were found to predict ITN ownership. Additional File 5 shows the adjusted odds ratio, 95% confidence intervals and p-values for these predictors.

A household with an under-five child was more than on-and-a-half times as likely to own an ITN as those without (OR, 1.55, CI, 1.53–1.94), while presence of health facility in the community and male gender of household head independently increased the odds of ITN ownership by about 100% (p < 0.0001 and 0.023 respectively). A unit increase in family size raised the odds of ownership by about 90% (OR, 1.88, CI, 1.13–3.13) and the presence of an educated woman in the household increased the odds by 36% (P = 0.012).

Interestingly, there was evidence for interaction between region and residence in relation to ITN ownership; being a rural household in the north significantly raised the odds of owning ITN about twice (p < 0.0001), even though residence in itself was not predictive. There was also evidence for synergism on a multiplicative scale between education and household wealth index on ITN ownership. A unit rise in wealth index when there was an educated woman in the household led to a 15% rise in the odds for owning a net (p = 0.025). There was, however, no evidence for interaction between combined wealth index and gender of household head.

For urban households, under-five child in the household and household religion were significant predictors of ITN ownership in the south after controlling for family size, education (using presence of an educated woman in the household as proxy), urban wealth index, and urban wealth index by household head's gender, household head's gender and health facility status. Only education was significantly predictive in the north and strongly so (OR, 2.05, CI, 1.18–3.55); however when an interaction term for education by urban wealth index was introduced into the model, while it did not change the picture in the south, it was significant in the north (OR, 1.33, CI, 1.02–1.75), such that in the final model, the effect of education alone on ownership of nets was slightly reduced, but was still significant (OR, 1.93, CI, 1.01–3.38).

Christianity increased the odds of ITN ownership about seven fold compared to Islam in the south (OR, 6.78, CI, 1.64–29.12) while the presence of an under-five child in the household increased the odds 3.4 times (OR, 3.42, CI, 1.92–6.40).

Similarly, ITN ownership in the south was also dependent on under-five child in the household and household religion, and rural wealth index in addition. Model III showed that ownership odds significantly increased 1.5 times with under-five in the household (p < 0.0001) and six-fold when household religion was Christianity compared to Islam, each variable adjusted for others in the model.

Religion was predictive of ITN ownership in the rural north and south. Just like it was for urban households in the south, in the rural south, the odds of ownership was significantly increased if the household was Christian (OR, 5.95, CI, 1.45–24.4) compared to if they were Muslim. However in the north, the relationship was in the opposite direction; Christian households had twice lowered odds of ITN ownership in this region compared to Muslim households, even after controlling for possible interaction between religion and wealth index. There was evidence for interaction between wealth index and household head's gender in the north; the odds of net ownership reduced by 30% with a unit rise in wealth index when the household head was female. Other religions were unimportant in determining net ownership in both regions.

#### Number of nets in the household

Overall, household ownership of any net was 23.9%, with 10.9% households owning more than one net. ITN ownership was however 10.1%, with 4.7% reporting ownership of more than one ITN. This means that of all households with nets, 42% had ITN while 58% had untreated bed nets; and of those who have more than one net, 43% were treated nets while 57% were untreated (Additional File 5). The mean number of nets per household reporting any net ownership was 1.82 (SD, 1.11) and 1.17 (SD, 1.25) for the total sample population.

#### Utilization of mosquito nets by under-five children

Overall, 11.5% (95% CI, 10.4%–12.6%) and 1.7% (95% CI, 1.3%–2.2%) of all eligible children slept under any net or ITN respectively, the night before the survey. Younger children (<2 years old) were more likely to be put under any net than older children although this was not significant for ITN. There was no association between the gender of the child and the use of nets (p = 0.36) however, region was significantly associated with utilization (p = < 0.0001); southerners were more likely to keep their children under nets than northerners, who were more likely to own nets. Utilization was commoner among rural children than urban children for any net but did not differ for ITNs (Additional File 6).

Education was very significantly associated with net utilization among under-five children (p = < 0.0001). The rate of utilization increased monotonously with level of education; those with higher education than secondary were about thrice as likely to put their under-five children under a net as the uneducated, and twice as much secondary school leavers.

In the combined data, household wealth index was significantly associated with utilization of any net and ITN by children under-5 (p = 0.004 and 0.003 respectively). Children who fell in the upper 2 quartiles are more likely to have slept under a net the night before the survey than the lower two quartiles. Those in the highest quartile are 1.5 times and 1.3 times as likely to use any net or ITN than those in the lowest quartiles respectively. However, when rural and urban dwellers were separated according to their wealth indices, utilization of nets was independent of the household wealth, although utilization still varied with residence by caregiver's level of education.

Additional File 7 shows utilization rates of ITN and any net for rural and urban under-five children by caregiver's level of education. Reported use of any net was significantly (p < 0.0001) higher among rural children of the less educated and uneducated caregivers (56%) than those whose caregivers were more educated (44%). However, the proportion of under-five children who used ITN among them was significantly (p < 0.0001) higher for caregivers with higher levels of education (secondary and higher, 56.7%).

In urban households, caregivers with secondary and higher levels of education were significantly (p < 0.0001) more likely to put their children under any net and were more likely to protect them with ITN, although this was not statistically significant (p = 0.146).

#### Utilization of any net

Multivariate analysis of utilization of any net by under-five children using the combined data showed that fever/convulsion in the previous two weeks, availability of health facility, residence and caregiver's level of education were predictors of utilization of any net, controlling for other variables in the model (Model 1, Additional File 8). Combined wealth index (CWI), child's age, family size, religion, region and region by residence, were not predictive of utilization of any net by under-five children.

If a child had fever/convulsion in the last two weeks, the odds of using a net the night before the survey was about 1.3 times higher than if fever/convulsion was not reported (p < 0.0001). This finding was similar for presence of a health facility in the community (OR, 1.29, CI, 1.01–1.63). In addition, the odds of an under-five child sleeping under any net the night before the survey was 40% higher if the caregiver was educated compared to uneducated caregivers (p = 0.016). There was also evidence for interaction between CWI and caregiver's level of education; for an educated caregiver, a unit increase in wealth index resulted in a 30% rise in the odds of utilization of any net (OR, 1.29, CI, 1.14–1.45) independent of other variables.

Under-five children who live in rural areas were about one-and-a-half times as likely to use any net as their counterparts in urban areas. When the data was split by urban or rural status of residence, using the combined wealth index as a measure of household wealth in both residences, (Models 2 and 3 respectively), fever/convulsion episode in the child and wealth index were found to be important predictors in the rural areas. The odds of utilization of any net was 1.5 times higher in children who had fever two weeks before the survey than under-five children who did not have fever (OR, 1.49, CI, 1.11–1.99); while wealth index independently increased the odds by 17% for every unit rise (OR, 1.17, CI, 1.01–1.37).

The presence of health facility in the community, caregiver's level of education and age of the child were found to predict utilization of any net in urban children. The odds of an under-five child using a net the previous night was 2.3 times higher for children who live in communities with health facilities than those without heath facilities (p = 0.001). An educated caregiver had a 2.16 higher odds of putting her child under a net than the uneducated, controlling for other factors (p = 0.008).

Caregiver's education was also found to interact with wealth index; the odds of net use increased by 30% when caregiver was educated (OR, 1.3, CI, 1.05–1.57) per unit rise in wealth index compared to an uneducated caregiver. Children less than two years of age were almost twice as likely to be put under a net as children between two and five years (OR, 0.56, CI, 0.36–0.85). When the effect of urban and rural wealth indices were examined with separate models developed for urban and rural children, the predictors were overall similar to those found using the combined wealth index. However, the age of the child was not predictive in urban region and fever was not significant for rural children.

#### Utilization of ITN

Additional File 9 shows the variables in the final logistic regression models developed to predict the use of ITN by under-five children. Only valid cases (2009) were included in the analysis. In the combined data (Model X), presence of health facility in the community where a child lived strongly predicted the use of ITN the night before the survey, with an odds three times higher than where health facilities were absent (OR, 2.93, CI, 1.29–6.78). Among Christian caregivers, the odds of an under-five child sleeping under an ITN the night before the survey was more than three times higher than for Muslim caregivers. When a caregiver was educated, a unit rise in wealth index increased the odds of utilization of ITN by 43%, other factors controlled for.

Age, family size, combined wealth index, residence, region, fever/convulsion episodes and caregiver's level of education were not found to independently predict utilization of ITN by under-five children in the combined data. However, when spilt by residence and output for the combined model generated for urban and rural communities, region was predictive in urban areas (Model Y) while there was no variable in the final model for rural communities. Children living in the north had five-fold lower odds of sleeping under an ITN than children living in the south (OR, 0.18, CI, 0.06–0.54).

Using rural and urban wealth indices, separate models were used to check for predictors. These models showed the same result for urban communities with region predicting use of ITN; while an interaction was found between rural wealth index and caregiver's level of education. When a caregiver in a rural area was educated, a unit increase in rural wealth index raised the odds of an under-five child sleeping under an ITN by 57% compared to an uneducated caregiver (OR, 1.57, CI, 1.06–2.32).

#### Utilization of nets and fever and/or convulsion prevalence

Overall fever/convulsion prevalence among under-five-children who slept under any net was 1.2 times greater than those who did not use any net; and more than one-and-a-half times greater in those who used ITN the night before the survey (Additional File 10).

There was a positive association between fever episodes in the last two weeks and the use of nets in under-five-children. The odds of using any net the night before the survey were about one-and-a-half times higher in children who had fever and/or convulsion in the last two weeks than children who had no fever and/or convulsion; and this was significant (p = 0.013). The difference was explained by the use of ITN; children with history of fever were twice as likely to have been put under an ITN the previous night as those with no such history, p = 0.006, while there is no significant difference among children who used other nets.

Among all children who used any net, those with history of fever and/or convulsion were 1.7 times more likely to use an ITN than an ordinary net; this was however not statistically significant. Usage of other nets was independent of fever history. Users and non-users had a prevalence of roughly 30%.

## Discussion

With data collected during a national household survey in October 2005, the set year for the achievement of the Abuja targets and mid-term assessment of the Abuja declaration, this study has demonstrated that Nigeria, though it has made some progress, is still very far from achieving the Abuja targets with regard to ITN coverage of the vulnerable groups.

### Household net ownership

Overall household ownership of any net was 23.9% (95% CI, 22.8%–25.1%) and ITN was 10.1% (95% CI, 9.2%–10.9%). Given the fact that ITNs were barely existent in the country before the launching of the RBM programme in Nigeria (coverage was 0% as reported by NetMark), these figures represent non-negligible progress. These results show about 100% improvement over the figures reported for any net and five-fold increase for ITN from the National Demographic Health Survey (NDHS) conducted in 2003 in the country; which reported 12% of the population owned any net and 2% owned ITN [31].

In 2004, NetMark reported 27% for ownership of any net and 9% for ITN, even though the study was done one year later than the NDHS. The exclusion of households without under-five children, which are less likely to have nets, could have caused an overestimation of the proportions and probably explains the higher proportion of ownership of any net compared to the current study. However, the higher ITN coverage in this study could reflect the massive distribution of free ITNs embarked upon by the National Malaria Control Programme (NMCP) in the last two years before the survey [45]. Although household ownership of ITN has increased in the Nigerian population, it is still low compared to other African countries, like Senegal, Malawi and Eritrea, which is the only country that has achieved the Abuja Targets [2].

The relatively higher proportion of net ownership in the rural area shows an existing demand in these areas and forms a good entry point for introduction of ITN since possession is the first step to utilization.

Although ITN ownership was not found to differ significantly by wealth index, the fact that the possession of any net varied by household wealth, with the rich more likely to own any net than the poor, may be a better indicator of the relationship between wealth and net ownership. There was no information on how the nets in the study were acquired, whether they were received free from the National Malaria Control Programme (NMCP) or purchased. However since the nets distributed free of charge to the vulnerable groups by NMCP (through health facilities and campaigns) were ITNs, this could account for the equity in ownership of ITN since those with ordinary nets must have paid for them. The correlation between presence of a health facility in the community and under-five child in households with ITN, also raises the probability of having received them from the programme, as this was one of the distribution channels.

This finding further highlights poverty as a potential barrier to scaling up of ITN in Nigeria as documented by other studies [26] and raises questions about funding large-scale distribution and the sustainability of such measures.

Two main strategies have been suggested to improve ITN coverage, forming two poles. Some authors [46,47] argue that to maintain a balance between equity and sustainability, the public sector should target subsidies at the vulnerable groups while the private sector is given room to grow. On the other hand, some believe ITN should be treated as a public good and should therefore be given out free of charge to everyone, especially the poor, who are most vulnerable to the disease; they argued that the international community can afford the cost for SSA [48].

The two positions are however valid. A large proportion of the population still live under a dollar per day and the vulnerable groups may not be able to pay for ITNs, even when subsidized. On the other hand, the limitedness of resources, considering the huge population of the country, makes free distribution unsustainable without guaranteed continuous support from donor agencies.

A recent study showed that an average Nigerian household was willing to pay Naira 7,324 (USD 61) per month for the control of malaria, an amount that was 37% higher than the current expenditure on malaria in form of protection, treatment and indirect costs [49] Mokuolu *et al*, in another study on patterns of consumption of the new and more expensive artemisinin-based combination treatment (ACT), reported a rise in the demand for the more expensive drug as a result of increased awareness about the efficacy of the medicine and the ineffectiveness of the cheaper alternative [41].

While for some, willingness to pay may match actual ability to pay, many may not be able to afford payment even with a high perception of risk. A window of opportunity for social marketing and commercial sales exists on one hand, while, on the other hand, it is also expedient for the most vulnerable groups to be provided for, so that ITN coverage can be scaled up and the RBM goal achieved [50].

Wealth index interacted significantly with presence of an educated eligible woman in the household (which was also an independent factor in the total population), underscoring the importance of education in ITN possession. Education as a determinant of net ownership has also been documented by a study in The Gambia that modeled the determinants of bed net ownership and the factors that influence the number of nets purchased [25].

This shows a cross-linkage between achieving the Abuja targets and other millennium development goals and it underscores the integration of the programmes targeted at achieving these goals. The relationship between religion and household net ownership points to the role of religious leaders in the propaganda for net ownership.

### Utilization of nets

Proportion of under-five children using ITN is still very low, although the rate found in this study – 1.7% (95% CI, 1.3%–2.2%) – was higher than the 2000 baseline of 0% and the 2003 NDHS figure of 1.2%, considering the 60% set as the Abuja target. There is an urgent need for the NMCP to intensify its efforts.

This study demonstrated higher utilization rates for any net among rural children than urban children. This was contrary to what was found in a meta-analysis of 13 surveys, which included five Demographic Health Surveys for African countries, where children in urban households were found to be more likely to use nets than children in rural households [21]. A similar meta-analysis carried out on studies conducted between 1999–2002 also reported the same findings [51]

However, these studies analysed surveys that were done as baseline assessment before the full-scale implementation of the RBM programme. There findings are, therefore, not likely to reflect the state of the art with utilization of nets.

Although use of ITN is low, the higher rates of use of ordinary nets show an existing culture of net use and represent an entry point for scaling up ITN. A massive net (re)treatment campaign should be embarked upon to turn these nets into ITN. Utilization was disproportionate to household net ownership. The north-south divide of this scenario particularly raises questions about the factors that could be responsible for this. Koreromp *et al *also found this gap between ownership and possession, and noted that nets were less likely to be used during hot and dry seasons [21]. However, this study was conducted during the rainy season when malaria transmission peaks and net utilization were supposedly high. Binka *et al *in a study of acceptability of nets in Ghana, found that 99% used their nets during rainy season [27]. The low rate of use by this vulnerable group therefore raises concern. Could it be that they are not given priority in the household for using the nets as there is scarcity of ITNs in the home? This is possible considering that the ratio of mean family size to mean number of ITNs per household was 4.5 ± 3.0:1 among families with ITN; however, since it is possible for more than one person to share a net and information was unavailable on this, it is difficult to ascertain this. Could it also be that caregivers are unaware of the need for consistent use or could the perception of risk be lower in the north than the south?

Educated caregivers with an increasing wealth index had higher likelihood of putting their children under ITNs than uneducated ones, even with increase in wealth index. This pre-supposes that, given equity in ownership of nets, there still remains a gradient between those children whose caregivers are educated and those who are not. This is an important finding that calls for a paradigm shift in the malaria control efforts since actual protection of these children depends on the use of the nets rather than ownership. The fight against malaria cannot be left only in the hands of the NMCP; it calls for a multi-sectoral approach involving, for instance, the Ministry of Education and the Ministry of Women Development.

The correlation between the presence of health facilities in the community and utilization of ITN, as has been highlighted above, could be due to the fact that these are ITN distribution outlets for the NMCP and could point to the effectiveness of this mechanism in reaching the vulnerable groups. However, the fact that this did not predict use when data was split by urban and rural residence calls for further analysis in this area. The north-south divide in under-five utilization of ITN also calls for further research into the possible barriers to use. Could it reflect cultural or religious reasons? This arises especially as Christians were more than three times more likely to put their children under ITNs than Muslims in the total population.

### Limitations of the study

In interpreting the results of this study, certain issues must be borne in mind. The sampling methodology used in this study (cluster design) is fraught with the problem of high intra-class correlation and taking outcomes to the exact percentage point may be misleading. However, since a large number of clusters were studied in this survey, this effect will be minimal.

Also, the survey questionnaire was not translated into local languages, the interviewers interpreted them to the respondents who did not understand English. It is possible for interviewers to misinterpret questions or introduce personal preferences. However, because they were all trained and the hypothesis of this study was generated after data collection, this should probably not have biased the study in a significant way.

The household participation rate in the study was 78%. Two zones (NCZ and SWZ) were overly represented in the sample, contributing over 20%, while two other zones contributed less than 15% to the sample. Differences between these zones could affect the parameters measured in this study, the direction of which cannot be ascertained as no information was available about those who did not participate in the study. Nevertheless, since most states in the south are similar and the same in the north, and the north to south ratio of the sample was about 1:1, this limitation may not adversely affect the study.

Although the PCA method used in developing the household wealth index only measures the long-term household wealth and does not account for short-term wealth or shocks to the household as other methods like income and expenditure do; this study was not particularly focused on the current resources available to households but on the class to which they belong based on their durable possessions and in relation to other households. This is therefore not likely to affect the socio-economic classification. Moreover, data on validation of the reported assets was not available in this study, thus this could be a potential limitation, however, some confidence in the result can be drawn from the fact that the findings are similar to what other studies have documented with regards to net ownership.

The use of an educated eligible woman in the household as a proxy for educational status of the household could introduce bias in the result. If there was more than one eligible woman in the house and the educated one happens not to have any under-five child, then she would not have been interviewed according to the study protocol, but since this would have been a non-differential misclassification, it is likely to have caused an underestimation of the odds of net ownership.

The use of fever as proxy for malaria also calls for caution in interpreting these results. Being a symptom for many other childhood illnesses with shared risk factors for malaria, like age, it is probable that children with diseases other than malaria could have been counted as malaria cases since no specific diagnosis was done to ascertain the cases. However, fever is the commonest symptom of malaria and 60% of every fever episode among under-five children in childhood, especially in the rainy season, is likely to be due to malaria. Moreover, it has been documented that in malaria endemic regions like Nigeria, malaria increases the risk of other childhood diseases e.g., pneumonia, and frequently co-exist with them in a co-morbid state [52,53]

Since this study was done during the rainy season, during which malaria transmission peaks, children are more likely to have more episodes of malaria. Therefore, it is unlikely that this proxy will adversely affect the findings, and even if it does, the misclassification will be non-differential as the question about fever and net use were asked independently, and as such would underestimate the prevalence throughout.

Finally, since this study, for the most part was a retrospective evaluation of self-reported behaviour patterns, it was subject to recall bias, however as the time-period was short (two weeks) this bias is minimal.

## Conclusion

Over a five year period, Nigeria has succeeded in achieving only 2.8% of the 60% expected coverage for under-five children with insecticide-treated nets. Although this is a non-negligible achievement considering the baseline situation of 0%, this progress is much too slow, if the target set for 2010 are to be achieved and, therefore, puts a prerogative on a more concerted effort by the malaria control body.

Achieving the set goal requires focused and well-informed strategies that are based on scientific evidence generated from local circumstances. This study has identified key issues constituting impediments in the cogwheels of progress; while poverty militates against ownership of net, lack of education curtail its use. This presents the policy makers with the challenge of addressing these issues decisively, if the RBM goal, or indeed the MDGs will be met.

Considering the time constraints, the aim must be a rapid scale-up among the target groups, while not neglecting the other members of the population. A pluralistic approach to scaling up, in which several distribution and financing mechanisms are combined has been recommended; [47] this includes commercial sales of nets, social marketing, community-based distribution and targeted subsidies. However, the choice of strategy must be based on evidence of what works in the context of the country to ensure adaptability. These strategies must target not only increasing household ownership but also utilization, which is most important for epidemiologic impact.

In addressing poverty as a hindrance to ownership, the government must decide what is workable for it within the limits of its resources; whether to continue giving out the nets free of charge, if it can sustain it, or to highly subsidize the nets while continuing to use the MCH clinics as distribution outlets, and continuing the mass ITN distribution campaign. The most important consideration should be that cost must not be a barrier to access to nets for the vulnerable groups.

Other measures of reducing the cost of ITN should be explored to ensure that the rest of the population not covered by subsidy or free distribution can have access to the nets at an affordable cost. Seeking transfer of net manufacturing technology from a sister African country like Tanzania, and removing taxes and tariffs on netting materials and insecticides are some of these measures. These will create an enabling environment for, and encourage local manufacturing of nets, which not only has the potential to substantially reduce the market price of the nets, but also to create employment opportunities and, as such, alleviate poverty.

Evidence from this study shows that NMCP needs to reassess the communication strategies it has been using for net delivery and promotion in the last five years. If nets have been given out free-of-charge to under-five children for five years and the coverages are still low, then it raises questions about the kind of messages being delivered to the population and how well they understand them. While recognizing that NMCP cannot embark on adult education of the vast majority in the rural communities who are illiterate, it could liaise with the Ministries of Communication, Education and Women Development in developing and disseminating information on ITN use and benefits in the language that the audience best understand.

With the foregoing, it is paramount for the federal government to increase its health expenditure to 15% of GNP as agreed during the RBM summit by the African Heads of States. The currently poor spending on health (less than 5% of GNP) is too small if the country is to achieve a quick win over malaria and achieve the MDGs, to which malaria control is so intricately linked.

## Competing interests

The authors declare that they have no competing interests.

## Authors' contributions

OBO conceived the design, acquired the data, analysed and interpreted the results. MH contributed to the analysis and revised the manuscript while OS contributed to acquiring the data and proof read the manuscript.

## Supplementary Material

Additional file 1 Socio-demographic characteristics of study households by region. Click here for file

Additional file 2 Household ownership of mosquito nets by background characteristic. Click here for file

Additional file 3 Predictors of household ownership of any net. Click here for file

Additional file 4 Predictors of household ownership of ITN. Click here for file

Additional file 5 Net Ownership and Type of net by Number of nets in households. Click here for file

Additional file 6 Utilization of mosquito nets by children. Click here for file

Additional file 7 Proportion of rural and urban under-five children who used nets the night before the survey by caregiver's education level and type of net used. Click here for file

Additional file 8 Logistic regression models for prediction of use of any net by under-five children. Click here for file

Additional file 9 Logistic regression models for prediction of utilization of ITN by under-five children. Click here for file

Additional file 10 Prevalence of fever episodes among under-five children by utilization of net. Click here for file
