# Supplementary material for: Utilization of insecticide-treated nets by under-five children in Nigeria: Assessing progress towards the Abuja targets
Source: Malar J. 2008 Jul 30;7:145. doi: 10.1186/1475-2875-7-145 (PMC2543041; doi:10.1186/1475-2875-7-145)
Supplement: Additional file 1 — Socio-demographic characteristics of study households by region. [file 1475-2875-7-145-S1.pdf]

Socio-demographic characteristics of study households by region

Socio-demographic characteristics	Region													
	South west n=1175		South east n=803		South south n=757		North west n=948		North east n=738		North central n=1167		Total n=5588	
	n	%	N	%	n	%	n	%	n	%	n	%	n	%
Residence														
Urban	389	33.5	12	1.5	339	45.6	402	47.3	121	20.7	636	55.2	1899	36.0
Rural	772	66.5	774	98.5	405	54.4	448	52.7	464	79.3	517	44.8	3380	64.0
Total	1161	100.0	786	100.0	744	100.0	850	100.0	585	100.0	1153	100.0	5279	100.0
Health facility in community														
Present	555	48.6	646	82.7	522	70.0	469	57.1	391	66.8	549	47.7	3132	59.9
Absent	587	51.4	135	17.3	224	30.0	352	42.9	194	33.2	602	52.3	2094	40.1
Total	1142	100.0	781	100.0	746	100.0	821	100.0	585	100.0	1151	100.0	5226	100.0
Household wealth index (combined)														
Lowest	233	20.0	224	28.2	135	18.1	292	31.1	164	37.4	159	14.0	1207	23.1
2nd	244	21.0	159	20.0	146	19.5	218	23.2	169	38.5	270	23.9	1206	23.1
3rd	378	32.5	188	23.6	192	25.7	248	26.4	66	15.0	379	33.5	1451	27.8
Highest	308	26.5	224	28.2	274	36.7	181	19.3	40	9.1	324	28.6	1351	25.9
Total	1163	100.0	795	100.0	747	100.0	939	100.0	439	100.0	1132	100.0	5215	100.0
Urban household wealth index													*(n=1899)*	
Lowest	63	16.4	1	8.3	64	19.0	121	30.3	29	43.9	177	28.5	455	25.0
2nd	127	33.0	2	16.7	84	25.0	121	30.3	17	25.8	186	30.0	537	29.5
3rd	95	24.7	6	50.0	100	29.8	78	19.5	9	13.6	113	18.2	401	22.0
Highest	100	26.0	3	25.0	88	26.2	79	19.8	11	16.7	145	23.3	426	23.4
Total	385	100.0	12	100.0	336	100.0	399	100.0	66	100.0	621	100.0	1819	100.0
Rural household wealth index													*(n=3229)*	
Lowest	298	38.9	92	12.0	153	38.0	199	44.7	109	31.7	126	25.0	977	30.3
2nd	94	12.3	226	29.5	48	11.9	100	22.5	159	46.2	87	17.3	714	22.1
3rd	192	25.0	187	24.4	64	15.9	103	23.1	44	12.8	143	28.4	733	22.7
Highest	183	23.9	262	34.2	138	34.2	43	9.7	32	9.3	147	29.2	805	24.9
Total	767	100.0	767	100.0	403	100.0	445	100.0	344	100.0	503	100.0	3229	100.0
Household religion														
Islam	223	21.2	10	1.4	28	4.5	195	73.1	195	45.8	449	46.9	1529	100
Christianity	813	77.3	695	97.2	580	93.5	229	26.8	229	53.8	498	52	3044	100
Other	16	1.5	10	1.4	12	1.9	2	0.1	1	0.5	11	1.1	52	100
Total	1052	100	715	100	620	100	426	100	854	100	958	100	4625	100
Household head's														
• Gender: Male	1012	88.1	715	91.4	623	83.8	903	98.5	716	98.9	1104	96.8	5073	93.0
Female	137	11.9	67	8.6	120	16.2	14	1.5	8	1.1	37	3.2	383	7.0
Total	1149	100.0	782	100.0	743	100.0	917	100.0	724	100.0	1141	100.0	5456	100.0
• Age (years): Mean	42.3		40.4		42.8		38.6		38.7		44.9		41.8	
*SD	13.7		9.9		14.4		7.6		9.3		14.9		12.7	
• Family size														
Mean	4.7		5.4		5.4		6.5		5.1		5.4		5.4	
*SD	2.4		2.3		2.7		3.4		3.9		2.8		3.0	

*SD, Standard deviation
