# Supplementary material for: Utilization of insecticide-treated nets by under-five children in Nigeria: Assessing progress towards the Abuja targets
Source: Malar J. 2008 Jul 30;7:145. doi: 10.1186/1475-2875-7-145 (PMC2543041; doi:10.1186/1475-2875-7-145)
Supplement: Additional file 2 — Household ownership of mosquito nets by background characteristic. [file 1475-2875-7-145-S2.pdf]

<u>Household ownership of mosquito nets by background characteristic.</u>

Households with at least one and more than one mosquito net (treated or untreated), and households that have at least one or more than one Insecticide Treated Net (ITN), by background characteristic

Background characteristic	At least one net (n=1193) % (n)	RR	95% CI	P-value for χ^2	More than one net (n=543) % (n)	RR	95% CI	P-value for χ^2	At least one ITN (n=501) % (n)	RR	95% CI	P-value for χ^2	More than one ITN (n=235) % (n)	RR	95% CI	P-value for χ^2	Number of households* (n=5588)
Residence																	
Urban	19.9 (348)	1.00		<0.0001	6.4 (112)	1.00		<0.0001	9.4 (164)	1.00		0.657	3.4 (60)	1.00		0.037	1752
Rural	25.0 (769)	1.26	1.13-1.41		12.2 (374)	1.90	1.55-2.33		9.8 (300)	1.04	0.87-1.25		4.7 (144)	1.38	1.02-1.84		3076
Total	23.1 (1117)				10.1 (486)				9.6 (464)				4.2 (204)				4828
Household religion																	
Islam	26.5 (387)	1.00		0.001	46.6 (214)	1.00			10.9 (159)	1.00			6.5 (95)	1.00			1458
Christianity	22.8 (662)	0.12	0.10-0.13		8.4 (244)	0.18	0.15-0.21	<0.0001	9.8 (285)	0.11	0.09-0.13	0.084	3.6 (104)	0.18	0.13-0.24		2909
Other	8.0 (4)	0.33	0.13-1.22		0.2 (1)	0.73	0.13-29.12		0.2 (1)	0.55	0.10-21.67		0.0 (0)	∞	0.09-∞		50
Total	23.8 (1053)	1.00			10.4 (459)				10.1 (445)				4.5 (199)				4417
Region																	
South west	15.9 (182)	1.00		<0.0001	5.8 (66)	1.00		<0.0001	9.6 (110)	1.00		<0.0001	4.6 (53)	1.00		<0.0001	1144
South east	36.5 (261)	2.30	1.95-2.71		15.4 (110)	2.66	1.99-3.57		11.9 (85)	1.23	0.95-1.62		4.2 (30)	0.91	0.69-1.79		715
South south	10.8 (78)	0.11	0.53-0.87		2.8 (20)	0.48	0.29-0.78		4.0 (29)	0.42	0.28-0.62		0.3 (2)	0.72	0.01-0.24		723
North west	32.8 (287)	2.06	1.75-2.43		21.9 (192)	3.80	2.91-4.95		9.8 (86)	1.02	0.78-1.33		8.5 (74)	1.82	1.30-2.57		876
North east	41.1 (176)	2.59	2.17-3.08		21.0 (90)	3.65	2.71-4.91		24.7 (105)	2.55	2.00-3.25		9.6 (41)	2.07	1.40-3.06		428**
North central	19.0 (209)	1.19	1.00-1.43		5.9 (65)	1.03	0.74-1.43		7.8 (86)	1.02	0.78-1.33		3.2 (35)	0.69	0.45-1.04		1099
Total	23.9 (1193)				10.9 (543)				10.1 (501)				4.7 (235)				4985
Combined wealth index																	
Lowest	24.1 (271)	1.00		0.038	13.3 (150)	1.00		0.009	9.1 (102)	1.00		0.048#	5.7 (64)	1.00		0.141	1126
2nd Quartile	23.8 (275)	1.01	0.85-1.12		11.4 (131)	1.17	0.92-1.50		11.0 (127)	0.82	0.63-1.08		5.0 (58)	1.13	0.78-1.64		1154
3rd Quartile	21.6 (303)	1.12	0.94-1.32		9.3 (131)	1.43	1.12-1.82		8.7 (122)	1.04	0.79-1.37		3.8 (53)	1.50	1.03-2.21		1404
Highest	26.3 (342)	0.91	0.78-1.07		10.0 (130)	1.30	1.05-1.70		11.4 (148)	0.79	0.61-1.03		4.5 (59)	1.25	0.86-1.81		1298
Total	23.9 (1191)				10.9 (542)				10.0 (499)				4.7 (234)				4982
Urban wealth index(n=348)					(n=112)												
Lowest	19.8 (86)	1.00		0.003	7.4 (32)	1.00		0.154	7.8 (34)	1.00		0.038	2.8 (12)	1.00		0.019	435
2nd Quartile	16.8 (88)	1.18	0.87-1.60		4.8 (25)	1.50	0.89-2.70		8.6 (45)	0.91	0.57-1.50		2.9 (15)	0.97	0.41-2.21		525
3rd Quartile	17.7 (68)	1.12	0.80-1.54		5.7 (22)	1.28	0.72-2.32		8.3(32)	0.94	0.56-1.57		2.3 (9)	1.18	0.50-3.20		384
Highest	26.0 (106)	0.76	0.57-1.02		8.1 (33)	0.86	0.50-1.43		13.0 (53)	0.60	0.38-0.94		5.9 (24)	0.47	0.20-0.98		408
Total	19.9 (348)				6.4 (112)				9.4 (164)				3.4 (60)				1752
Rural wealth index (n=769)					(n=374)												
Lowest	18.1 (166)	1.00		<0.0001	10.6 (97)	1.00		0.017	7.2 (66)	1.00		<0.0001	5.1 (47)	1.00		0.122	917
2nd Quartile	30.1 (203)	0.60	0.49-0.74		14.8 (100)	0.71	0.53-0.95		13.5 (91)	0.53	0.38-0.74		5.9 (40)	0.87	0.56-1.35		675
3rd Quartile	24.4 (172)	0.74	0.60-0.92		10.2 (72)	1.04	0.76-1.43		8.9 (63)	0.81	0.56-1.16		4.1 (29)	1.25	0.77-2.05		705
Highest	29.1 (226)	0.62	0.51-0.76		13.4 (104)	0.79	0.59-1.05		10.1 (78)	0.72	0.51-1.01		3.5 (27)	1.47	0.90-2.46		776
Total	25.0 (767)				12.1 (373)				9.7 (298)				4.7 (143)				3073

*Analysis was done for households without missing data **Missing 42% of data P-value significant at <0.05 #P-value for Fisher's exact test
