# Supplementary material for: Utilization of insecticide-treated nets by under-five children in Nigeria: Assessing progress towards the Abuja targets
Source: Malar J. 2008 Jul 30;7:145. doi: 10.1186/1475-2875-7-145 (PMC2543041; doi:10.1186/1475-2875-7-145)
Supplement: Additional file 3 — Predictors of household ownership of any net. [file 1475-2875-7-145-S3.pdf]

Predictors of household ownership of any net

[$]Predictors	COMBINED Model A* (n=4186)[#] [**]*OR*	*95% CI[**]*	*P-value*
U5 child in The HH[++]			
No	1.00		
Yes	1.60	1.40–1.90	<0.0001
Family size[∞]	2.23	1.57-3.18	<0.0001
Residence			
Urban	1.00		
Rural	1.26	1.05-1.52	0.021
Education			
No	1.00		
Yes	1.29	1.08-1.53	0.005
Region*Residence			
South*Urban	1.00		
North*Rural	1.51	1.25-1.82	<0.0001
Combined WI	1.24	1.15-1.34	<0.0001

[$]Predictors	URBAN Model B§ South (n=589)[#]			North (n=891)[#]		
	^OR	*95% CI*	*P-value*	^OR	*95% CI*	*P-value*
Education						
No				1.00		
Yes	-	-		1.42	1.02-1.98	<0.0001
Urban WI	-	-	-	1.28	1.09-1.50	0.003
Health facility						
Absent	1.00			1.00		
Present	2.88	1.54-5.37	0.001	0.59	0.41 - 0.81	0.038
U5 child in the HH[++]						
No	1.00			-		
Yes	3.24	1.99-5.27	<0.0001	-	-	-

[$]Predictors	RURAL Model C† South (n=1587)[#]			North (n=1119)[#]		
	[†]OR	*95% CI*	*P-value*	[†]OR	*95% CI*	*P-value*
U5 child in the HH						
No	1.00			-		
yes	1.74	1.36-2.23	<0.0001	-	-	-
Family size[∞]	3.59	1.91-6.76	<0.0001	2.71	1.43-5.11	0.002
Health facility						
Absent	1.00			-		
Present	1.58	1.20–2.08	0.001	-	-	-
HH Religion						
Islam	1.00			1.00		
Christianity	2.35	1.32-4.20	0.004	0.41	0.30-0.55	<0.0001
Other	1.24	0.25-6.18	0.878	0.23	0.03-1.91	0.175
Rural WI	1.33	1.19-1.48				
Education						
No	1.00			-		
Yes	1.55	1.16-2.09	0.003	-	- -	-

[$] Variables in the final models. [#]Data included in the analysis, all missing data were excluded. P-values significant at 0.05 CI, confidence interval

[∞] log value was used in analysis H&L, Hosmer and Lemeshow goodness-of-fit test. [++]HH, households; WI, wealth index;

* Logistic regression run for combined data (H&L test P-value =0.82)

[**]OR, Odds ratio; adjusted for religion, region by residence, religion by region, and combined household wealth index by household head's gender.

§ Model developed for urban household, data split by region (H&L test P-value: South, 0.96; North, 0.32)

^OR, odds ratio adjusted for religion, family size, religion by region, education by urban wealth index and urban wealth index.

† Model developed for rural households, data split by region (H&L test P-value: South, 0.07; North, 0.34)

[†]OR, odds ratio adjusted for household head's gender, rural wealth index, and rural wealth index by household head's gender.
