# Supplementary material for: Utilization of insecticide-treated nets by under-five children in Nigeria: Assessing progress towards the Abuja targets
Source: Malar J. 2008 Jul 30;7:145. doi: 10.1186/1475-2875-7-145 (PMC2543041; doi:10.1186/1475-2875-7-145)
Supplement: Additional file 4 — Predictors of household ownership of ITN. [file 1475-2875-7-145-S4.pdf]

Predictors of household ownership of ITN

COMBINED Model I* (n=4186) #

$Predictors	**OR	95% CI**	P-value
U5 child in HH++			
No	1		
Yes	1.55	1.53 - 1.94	<0.0001
Health facility status			
No	1	1.59 - 2.53	<0.0001
Yes	1.97	1.55 - 2.50	
Region*Residence			
South*Urban	1		
North*Rural	1.97	1.55-2.50	<0.0001
CWI*Education			
CWI*No	1		
CWI*Yes	1.15	1.02 - 1.29	0.025
HH head gender			
Female	1		
Male	1.87	1.09 - 3.20	0.023
Family size	1.88	1.13 - 3.13	0.015
Education			
No	1		
Yes	1.36	1.07 - 1.73	0.012

URBAN Model II§

$Predictors	South (n=589)# ^OR	95% CI	P-value	North (n=1047)# €OR	95% CI	P-value
U5 child in HH						
No	1			-		
Yes	3.42	1.87 - 6.27	<0.0001	-	- -	-
HH Religion						
Islam	1		0.026	-		
Christianity	6.78	1.61 - 28.63	0.009	-	- -	-
Other	3.27	0.28- 41.20	0.353	-	- -	-
Health facility						
No	-			1		
Yes	-	- -	-	5.8	2.05-16.41	0.001
Education^^						
No	-			1		
Yes	-	- -	-	1.93	1.10-0.38	0.022
UWI* Education						
UWI*No				1		
UWI*Yes				1.33	1.02-1.75	0.039

RURAL Model III†

$Predictors	South (n=1587)# †OR	95% CI	P-value	North (n=1119)# $OR	95% CI	P-value
U5 child in HH						
No	1					
yes	1.47	1.001-2.15	0.05			
HH Religion						
Islam	1			1		
Christianity	5.95	1.45-24.4	0.013	0.43	0.29-0.64	<0.0001
Other	0.00	0.00	0.998	0.00	0.00	0.998
RWI	1.37	1.18-1.60	<0.0001			
Health facility status						
No	1					
Yes	3.16	2.10-0.76	<0.0001	-	-	-
RWI*HH head gender						
WI*Female	1					
WI*Male	0.7	0.55-0.89	0.001			

$Variables in the final model. #Data included in the analysis, all missing data were excluded from the analysis P-values significant at 0.05 CI, confidence interval H&L, Hosmer and Lemeshow goodness-of-fit test. ++HH, households; RWI, rural wealth index; RWI*HH head's Gender, interaction term for wealth index and household head's gender; Region*Residence, interaction term for dichotomised region and residence (urban/rural); CWI*Education, interaction term for combined wealth index and education; UWI* Education, interaction term for urban wealth index and education. Education^^, presence of an educated eligible female respondent in the household

* Logistic regression run for combined data (H&L test P-value =0.11) **OR, Odds ratio; adjusted for religion, residence, family size (log value was used in analysis), combined household wealth index, and combined household wealth index by household head's gender.

§ Model developed for urban household, data split by region (H&L test P-value: South, 0.17; North, 0.11) ^OR, odds ratio adjusted for family size, education (dichotomised), education by urban wealth index, urban wealth index, urban wealth index by household head's gender, and household head's gender. €OR, odds ratio adjusted for family size, U5 child in the household, religion, health facility status, urban wealth index, urban wealth index by household head's gender, and household head's gender

† Model developed for rural households, data split by region (H&L test P-value: South, 0.78; North, 0.16) †OR, odds ratio adjusted for household head's gender, rural wealth index by household head's gender, health facility status, education (dichotomised), education by rural wealth index, and religion. $OR, odds ratio adjusted for rural wealth index, U5 child in the household, education (dichotomised), education by rural wealth index, and household head's gender.
