# Supplementary material for: Utilization of insecticide-treated nets by under-five children in Nigeria: Assessing progress towards the Abuja targets
Source: Malar J. 2008 Jul 30;7:145. doi: 10.1186/1475-2875-7-145 (PMC2543041; doi:10.1186/1475-2875-7-145)
Supplement: Additional file 5 — Net Ownership and Type of net by Number of nets in households. [file 1475-2875-7-145-S5.pdf]

<u>**Net Ownership and Type of net by Number of nets in households**</u>

**Number of nets**	**Net Ownership**							
	Households with Any net						All Households	
	Treated		Untreated		Total		(n)	(%)
	(n)	(%)	(n)	(%)	(n)	(%)		
Only one net	266	5.3	384	7.7	650	13.0	4985	100
More than one	235	4.7	308	6.2	543	10.9	4985	100
At least one	501	10.1	692	13.9	1193	23.9	4985	100
