# Supplementary material for: Utilization of insecticide-treated nets by under-five children in Nigeria: Assessing progress towards the Abuja targets
Source: Malar J. 2008 Jul 30;7:145. doi: 10.1186/1475-2875-7-145 (PMC2543041; doi:10.1186/1475-2875-7-145)
Supplement: Additional file 6 — Utilization of mosquito nets by children. [file 1475-2875-7-145-S6.pdf]

Children under the age five years who slept under a mosquito net the night before the survey and children who slept under an Insecticide Treated Net (ITN), by background characteristics

Background characteristic	Children under age five years who slept under a net last night	P-value for χ^2	Children under age five years who slept under an ITN last night	P-value for χ^2	Eligible children
	% (n=411)		% (n=61)		(n=3585)
Age (in years)					
<1	11.5 (68)	0.029	1.7 (10)	0.296	589
1	14.5 (107)		2.6 (19)		740
2	10.9 (85)		1.8 (14)		780
3	12.2 (89)		1.4 (10)		731
4	9.1 (62)		1.2 (8)		685
Total	11.7 (411)		1.7 (61)		3525
Gender					
Male	11.9 (208)	0.36	1.8 (31)	0.58	1754
Female	10.9 (178)		1.5 (25)		1639
Total	11.4 (386)		1.7 (56)		3393
Region					
South west	12.5 (87)	<0.0001	2.4 (17)	0.037	698
South east	16.0 (107)		2.5 (17)		668
South south	8.3 (27)		.09 (3)		327
North west	10.8 (78)		1.9 (14)		721
North east	14.5(61)		1.4 (6)		422
North central	7.3 (51)		0.6 (4)		697
Total	11.6 (411)		1.7 (61)		3533
Residence					
Urban	9.9 (130)	0.026	1.8 (23)	0.468	1308
Rural	12.4 (259)		1.4 (30)		2082
Total	11.5 (389)		1.6 (53)		3390
Caregiver's education level					
None	8.9 (84)	<0.0001	1.0 (9)	0.0003	942
Primary	10.4 (106)		1.1 (11)		1022
Secondary	12.1 (128)		2.0 (21)		1062
Higher	25.3 (76)		4.3 (13)		300
Total	11.8 (394)		1.9 (45)		3326
Caregiver's religion					
Islam	10.2 (119)	0.12	0.7 (8)	0.005	1164
Christianity	12.7 (270)		2.2 (46)		2126
Other	4.5 (1)		0.0 (0)		24
Total	11.5 (390)		1.6 (54)		3314
Household wealth index (combined data)					
Lowest	9.3 (77)	0.004	1.5 (12)	0.003	824
2nd Quartile	12.9 (109)		1.9 (16)		847
3rd Quartile	10.1 (98)		0.7 (7)		970
Highest	14.3 (126)		2.9 (26)		884
Total	11.6 (410)		1.7 (61)		3524
Urban household wealth index	(n=130)		(n=23)		(n=1308)
Lowest	8.7 (32)	0.399	1.4 (5)	0.108	367
2nd Quartile	9.3 (37)		1.3 (5)		398
3rd Quartile	10.0 (25)		3.6 (9)		249
Highest	12.5 (36)		1.4 (4)		287
Total	10.0 (130)		1.8 (23)		1301
Rural household wealth index	(n=259)		(n=30)		(n=2082)
Lowest	10.4 (56)	0.331	1.3 (7)	0.08	536
2nd Quartile	12.8 (71)		1.6 (9)		553
3rd Quartile	12.2 (59)		0.4 (2)		482
Highest	14.1 (72)		2.4 (12)		510
Total	12.4 (258)		1.4 (30)		2081
