# Supplementary material for: Utilization of insecticide-treated nets by under-five children in Nigeria: Assessing progress towards the Abuja targets
Source: Malar J. 2008 Jul 30;7:145. doi: 10.1186/1475-2875-7-145 (PMC2543041; doi:10.1186/1475-2875-7-145)
Supplement: Additional file 7 — Proportion of rural and urban under-five children who used nets the night before the survey by caregiver's education level and type of net used. [file 1475-2875-7-145-S7.pdf]

Proportion of rural and urban under-five children who used nets the night before the survey by caregiver's education level and type of net used.

**Caregiver's education level**	**Residence**			
	Rural		***Urban***	
	Any net (n=259)	*ITN (n=30)*	*Any net (n=130)*	*ITN (n=23)*
	% (n)	% (n)	% (n)	% (n)
None	25.0 (63)	16.7 (5)	13.6 (17)	14.3 (3)
Primary	31.0 (78)	26.7 (8)	20.0 (25)	14.3 (3)
Secondary	24.2 (61)	26.7 (8)	48.0 (60)	54.4 (11)
Higher	19.8 (50)	30.0 (9)	18.4 (23)	19.0 (4)
Total	100 (252)	100 (30)	100 (125)	100 (21)
p-value for χ^2	***<0.0001***	***<0.0001***	***<0.0001***	***0.146***
