# Supplementary material for: Utilization of insecticide-treated nets by under-five children in Nigeria: Assessing progress towards the Abuja targets
Source: Malar J. 2008 Jul 30;7:145. doi: 10.1186/1475-2875-7-145 (PMC2543041; doi:10.1186/1475-2875-7-145)
Supplement: Additional file 8 — Logistic regression models for prediction of use of any net by under-five children. [file 1475-2875-7-145-S8.pdf]

Logistic regression models for prediction of use of any net by under-five children

*Model 1 (Combined) (n=3163)				+Model 2 (Urban) (n=1967)				++Model 3 (Rural) (n=1172)			
**Variables**	**OR	95% CI**	#P-value	**Variables**	OR	95% CI	#P-value	**Variables**	OR	95% CI	#P-value
Fever/convulsion episode				*CWI*Caregiver's education*				*Fever/convulsion episode*			
No	1.00			CWI*None	1.00			No	1.00		
Yes	1.28	1.14 -1.45	<0.0001	CWI*Educated	1.30	1.05-1.57	0.017	Yes	1.49	1.11-1.99	0.008
Health facility				*Health facility*							
Absent	1.00			Absent	1.00			***CWI*	1.17	1.01-1.37	0.038
Present	1.29	1.01 - 1.63	0.039	Present	2.26	1.39-4.65	0.001				
Caregiver's education				*Caregiver's education*							
None	1.00		0.016	None	1.00						
Educated	1.40	1.06- 1.84		Educated	2.16	1.22-3.81	0.008				
*CWI*Caregiver's education*				*Age in years*							
CWI*None	1.00			Less than 2	1.00						
CWI*Educated	1.29	1.14-1.45	<0.0001	>2 and < 5	0.56	0.36-0.85	0.007				
Residence											
Urban	1.00										
Rural	1.43	1.12 -1.82	0.004								

Missing data excluded from all analysis. ** OR, adjusted odds ratio; CI, 95% confidence interval #P-value for Wald statistic **CWI, combined wealth index CWI*Caregiver's education, interaction term for combined wealth index and caregiver's education

*Model 1: for combined data; adjusted for child's age, family size, religion, region (dichotomised), region by residence, and combined wealth index (as a continuous variable); Hosmer & Lemeshow test for goodness-of-fit =0.225

+Model 2 for urban children; adjusted for family size, region (dichotomised), religion, fever/convulsion episode, and combined wealth index (as a continuous variable). H&L test for goodness-of-fit= 0.72

++Model 3 for rural children also adjusted for age, family size, region (dichotomised), religion, caregiver's education, and combined wealth index by caregiver's education H&L test for goodness-of-fit =0.23
