# Supplementary material for: Utilization of insecticide-treated nets by under-five children in Nigeria: Assessing progress towards the Abuja targets
Source: Malar J. 2008 Jul 30;7:145. doi: 10.1186/1475-2875-7-145 (PMC2543041; doi:10.1186/1475-2875-7-145)
Supplement: Additional file 9 — Logistic regression models for prediction of utilization of ITN by under-five children. [file 1475-2875-7-145-S9.pdf]

Logistic regression models for prediction of utilization of ITN by under-five children

*Model X (Combined) (n=2009)				+Model Y (Urban) (n=743)			
**Variables**	**OR	95% CI**	#P-value	**Variables**	##OR	95% CI	#P-value
Health facility				*Region*			
Absent	1.00			South	1.00		
Present	2.95	1.29-6.78	0.03	North	0.18	0.06-0.54	0.002
Religion							
Islam	1.00		0.048				
Christianity	3.30	1.28-8.53	0.014				
Other	0.00	0.00	0.999				
*CWI*Caregiver's education*							
*CWI*None*	1.00						
CWI*Educated	1.43	1.04 -1.98	0.03				

*Model X: for combined data; **OR, odds ratio adjusted for child's age, family size, region (dichotomised), region by residence, residence, fever/convulsion episode, and combined wealth index (as a continuous variable); Hosmer & Lemeshow test for goodness-of-fit =0.09.

+Model Y: for urban residence, ##OR, adjusted for child's age, family size, region by residence, residence, fever/convulsion episode, and combined wealth index (as a continuous variable), health facility status, religion and combined wealth index by caregiver's education.

*CWI*Caregiver's education*: interaction term for combined wealth index and caregiver's education.
