# Supplementary material for: Utilization of insecticide-treated nets by under-five children in Nigeria: Assessing progress towards the Abuja targets
Source: Malar J. 2008 Jul 30;7:145. doi: 10.1186/1475-2875-7-145 (PMC2543041; doi:10.1186/1475-2875-7-145)
Supplement: Additional file 10 — Prevalence of fever episodes among under-five children by utilization of net. [file 1475-2875-7-145-S10.pdf]

Prevalence of fever episodes among under-five children by utilization of net.

**Use of net**	**Child had fever in the last 2 weeks**								
	Yes		No		Total		*OR	95% CI**	p-value
	n	%	n	%	n	%			
Under-5 used any net									
Yes	133	32.4	276	67.6	411	100			
No	829	26.6	2293	73.4	3122	100	1.32	1.05 - 1.66	0.013
Total	962	27.2	2571	72.8	3533	100			
Under-5 used ITN									
Yes	26	42.6	35	57.4	61	100			
No	936	27.0	2536	73.0	3472	100	2.01	1.16 - 3.46	0.006
Total	962	27.2	2571	72.8	3533	100			
Under-5 used ITN									
Yes	26	42.6	35	57.4	61	100			
No	107	30.6	243	69.4	350	100	1.69	0.97 - 2.94	0.063
Total	133	32.4	278	67.6	411	100			

*OR, Odds ratio **CI, confidence interval
